# Predictive Value of Marker of Proliferation Ki-67 and Cell Cycle Dependent Protein kinase Inhibitor P16^INK4a^ in Cervical Biopsy to Determine Its Biological Behaviour

**DOI:** 10.31557/APJCP.2021.22.7.2237

**Published:** 2021-07

**Authors:** Usha Sarma, Gokul Chandra Das, Bidula Sarmah

**Affiliations:** 1 *Department of Pathology, Gauhati Medical College, Guwahati, India. *; 2 *Department of Obstetrics and Gynecology, Gauhati Medical College, Guwahati, India. *; 3 *Department of Education, Gauhati University, Guwahati, India. *

**Keywords:** CIN, cervical dysplasia, immunohistochemistry, cervical cancer

## Abstract

**Objective:**

The aim of the study is to analyse the Immuno-histochemical expression of Ki-67 and P16^INK4a^ in CIN and cervical cancer cases and their utility to determine the accuracy of histological diagnosis and prediction of biological behavior of cervical lesion.

**Methodology:**

A retrospective cross-sectional study was carried in 110 numbers of cervical biopsy that included 25 CIN1, 21 CIN2, 12 CIN3, 26 SCC and 01 adenocarcinoma and 25 non neoplastic lesion. The tissue sections were stained with Ki-67 and P16^INK4a^.

**Results:**

Ki-67 expression was seen in 55.5% (61/110) cases of cervical lesion., out of which 3.6% (4/110; cervicitis -2/110 and metaplasia-2/110) cases were non dysplaia, 51.8% (57/110) cases were dysplasia /CIN of varying grade including invasive cancer. P16^INK4a^ expression was noted 51.8% (57/110). There was an increasing trend of the intensity of Ki-67 and P16^INK4a^ from focal positivity in low grade lesion to diffuse intensity in higher grade lesion and is statistically significant. There was strong association between the two variables Ki-67 and P16^INK4a^ positive cases with their histologic grade.

**Conclusion:**

Though histopathology remains the ‘‘gold standard’’ for the diagnosis of CIN, both low and high-grade, biomarkers like Ki-67 and P16^INK4a ^have emerged as helpful adjuncts. Their combined use may assist in the histopathologic classification of preinvasive lesions and facilitate the distinction from nondysplasia.

## Introduction

Cervical intraepithelial neoplasia(CIN) is a term that describes a continuum of cervical change that begins with mild dysplasia and ends with invasive cancer after passing progressively through the intermediate stages of intraepithelial disease (Richart, 1987). In CIN1 (mild dysplasia), neoplastic basaloid cells occupy the lower third of the epithelium; in CIN2 (moderate dysplasia), neoplastic basaloid cells and mitotic figures occupy the lower two-thirds of the epithelium; and in CIN3, mitotic figures and basaloid cells are found throughout the whole thickness of the epithelium. CIN1 develops as a result of a productive HPV infection, is designated low-grade CIN and regress spontaneously within one or two years. Due to high-risk HPV infection, the cytological change may persist and these lesions contain more severe cytological atypia (CIN2 and CIN3), which are thought to be true potential precursors of cancer designated as high-grade CIN. Though histology is the gold standard for morphological change of cervical epithelium, there may be intraobserver and inter-observer variability from the part of the pathologist that the lesion being diagnosed is a true precursor of cancer or not. Since CIN is a dynamic process (not a static process), that can progress or regress, the conventional haematoxylin and eosin (H & E), gives a false impression of a static process. These points emphasize the need to identify and discover new markers that help in distinguishing CIN from other benign conditions and establish it as a dynamic process. Several immunohistochemical markers determine the proliferating nature of a lesion. Several studies state that P16 INK4a and Ki-67 may have a potential role in predicting the nature of CIN in progression to cervical cancer (Yu et al., 2016; Sharon et al.,2016, Ahmed SA et al.,2017, Hebbar et al., 2017). Cell cycle-dependent protein kinase inhibitor P16^INK4a^ is a protein that can negatively regulate the cell cycle. HPV persistent infection causes overexpression of P16 (Kim et al., 2005), The application of both P16 and Ki-67 is helpful to identify patients with a high risk of SCC; thereby reduces the over/under-diagnosis and provides high value for the differential diagnosis of young women with SCC and Cervical Intraepithelial lesion (Qin et al.,2019). A combination of both P16^INK4a^ and Ki-67 immunohistochemical markers were applied to post-therapy cases of cervical cancer to detect residual tumour by Desai et al., 2021 with an accuracy of 82.86% (95%CI: 66.4- 93.4) for P16^INK4a^ and 100% (CI:90.0-100.0) for Ki-67 stain. (Desai et al., 2021). Immunostaining of P16^INK4a^ allows precise identification of even small focus of CIN or cervical cancer lesions in biopsy sections and helped reduce inter-observer variation in the histopathological interpretation of cervical biopsy specimens (Doeberitz et al., 2001). K i-67 is a proliferation marker that is confined to the parabasal cell layer of normal stratified squamous mucosa. It is a nuclear and nucleolar protein expressed only in active phases and cell cycle (G1, S, G2, and M phases) but not in resting phases (G0 and early G1). Overexpression of Ki-67 correlates with high cellular proliferation. Since HPV infection leads to increased epithelial cell proliferation in infected tissue, increased Ki-67 staining can be an indicator of HPV (Conesa-Zamora et al., 2009a; Bruni et al., 2019). As a proliferative marker, Ki-67 can be used to see its expression in cervical biopsy tissue with their histologic grade. Ki-67 is an antigen expressed in the nuclei of proliferating cells, has also been studied as an indicator of CIN. Ki-67 is expressed in the nucleus during the whole cell cycle, except for the G0 and G1 early phases. This protein has a function of growth in the human tumour, and expression of its marker could suggest the degree of malignancy (Kruise et al., 2004; Haris et al., 2004; Cambruzze et al., 2005). Therefore, Ki-67 may be a useful marker of proliferation in dysplastic lesions, particularly in CIN and, in addition, can be of prognostic value (Goel et al., 2005). Being a proliferative marker, Ki-67 may be expressed in benign lesion undergoing reparative change and the use of this marker singly might give a false impression of the true nature of the lesion. In problematic cases, Ki-67 alone cannot differentiate between dysplasia and benign reactive change (Asani et al., 2013). Supplementary use of p16 staining along with Ki-67 significantly improves the accuracy of grading CIN lesions by a single pathologist, equivalent to an expert consensus diagnosis. Even a study by Zummeren et al., (2018) reports that grading of cervical intraepithelial neoplasia (CIN) by a Ki-67 and p16^ink4a^ immunoscore system has higher accuracy and reproducibility compared with current CIN grading. By use of the Ki-67 and p16^ink4a^ immunoscore, most classical CIN2 can be divided into more accurately graded CIN1 and CIN3 (Marjolein V Zummeren et al., 2018).

Hence the study has been carried out with the following objectives: (1). To evaluate the expression of p16 INK4a and Ki-67 in cervical intraepithelial neoplasia (CIN) and cervical cancer and (2) To study their utility to determine the accuracy of histological diagnosis and prediction of biological behaviour of cervical lesion.

## Materials and Methods

It is a retrospective study of Ki-67 and P16^INK4a^ expressions on cervical tissue diagnosed as CIN of varying grade and cervical cancer using the immunohistochemical method. The study has been ethically permitted by the institutional ethics committee vide no 233/2013/252. The study included 110 numbers of paraffin-embedded cervical biopsy tissue blocks (either wedge/punch cervical biopsy/ hysterectomy) collected from the archive of the Pathology Department, Gauhati Medical College for the period of 2 years from June’ 2014 to May’2016. Out of 110 cases, 68 cases had an initial diagnosis of CIN of varying grade and 27 had cervical cancer ( SCC-26 and adenoca-1); 10 cases had chronic cervicitis and 5 had metaplastic change. Endocervical polyps were excluded. All the paraffin-embedded tissue blocks were cut at 4 μM thickness and two sections were made ready from each block. One tissue section was placed on albuminized slides for routine hematoxylin and eosin stain and the other set of tissue sections was mounted on poly L –lysine coated slides for immunostain. The poly L –lysine coated slides were placed in the oven for 10 minutes. Sections were then deparaffinized by passage through xylene and subsequently rehydrated in graded alcohol of decreasing concentration i.e. 100 %, 70% and 50% at five minutes interval per change. They were then rinsed in running water for five minutes. Antigen retrieval, in which the sections were placed in the target antigen retrieval buffer solution was performed using EZ-Retriever system v.2 ( Biogenex) at temperature 60^o^C for five minutes one cycle followed by setting the temperature at 90^o^C for five minutes two cycle. Then the sections were allowed to cool at room temperature for 20 minutes. They were washed with running water and then rinsed with tris-buffer saline (TBS). Sections were incubated for 30 minutes at room temperature with mouse monoclonal anti- p16 INK4a antibody (Ready to use, from BioGenex, Fremont, CA) in one slide and Ki-67 (SP6) rabbit monoclonal antibody (Ready to use from CELL MARQUE, Rocklin CA) in the other slide. After washing thoroughly with TBS at pH7.4, high definition amplifier was added to the slides and incubated for 30 minutes at room temperature in a humidity chamber, followed by rinsing with TBS. High definition polymer HRP Label (secondary antibody) is added to the slides and incubated for 30 minutes at room temperature. Again the slides were washed with TBS at PH7.4. A drop of diaminobenzidine (DAB) was then spread over the sections for seven minutes and then it was rinsed in water. The sections were counterstained with haematoxylin for 30-45 seconds before rinsing with running water for three minutes and dehydrated in increasing alcohol concentration and mounted. All the staining pattern were compared with the result of positive control and negative control in each set of immuno-stain. 

The slides were examined with the help of an Olympus microscope under 10X and 40 X power eyepiece and scored all immunohistochemical stains in the epithelium of each biopsy. The scoring of p16 generally includes both nuclear and cytoplasmic staining, graded as 0 (no staining), 1 (rare singly dispersed cells staining), 2 (patchy but strong staining, often not continuous from basement membrane), and 3 (strong and diffuse staining, usually continuous staining from the basement membrane and extending upward in proportion to lesion grade) (Galgano et al., 2010) 

To determine the grade of Ki-67 expression, nuclei of 200 epithelial cells located across the whole epithelial layer has been examined in a high-power field. The ki-67 index is defined as the percentage of Ki-67 positive cells. It will be Graded as 1+ (5%), 2+ (5-30%), and 3+ (>30%) depending upon the percentage of Ki-67 positive cells (the Ki-67 index) as stated by Galgano et al., 2010. The scoring of Ki-67 includes nuclear staining only, and scored as 0 (no staining), 1 (1-2 layers of basal/parabasal staining), 2 (diffuse staining confined to the bottom third or superficial staining but with skip areas usually between parabasal and upper zones), and 3 (continuous staining of greater than the lower third of the epithelium). 

*Statistical Analysis*

The patients’ data were collected from the medical records department and data entry was carried out by using MICROSOFT OFFICE EXCEL 2007. The associations between variables (The expression of P16^INK4a^ and histological diagnosis of cervical tissue) was calculated by using the Chi-Square (*χ*^2^) test of significance; P values less than 0.5 was considered statistically significant. The standard methods of statistical analysis were performed by adopting the statistical software Graph Pad InStat. The sensitivity and specificity with predictive value were calculated out by applying standard formulae (Lalkhen and McCluskey., 2008) 

The sensitivity of a clinical test refers to the ability of the test to correctly identify those patients with the disease.

Sensitivity = True positives / (True positives + False negatives). 

The specificity of a clinical test refers to the ability of the test to correctly identify those patients without the disease. 

Specificity = True negatives/ (True negatives + False positives). 

The PPV of a test is a proportion that is useful to clinicians since it answers the question: ‘How likely is it that this patient has the disease given that the test result is positive?’

Positive predictive value =True positives / (True positives + False positives). 

Negative predictive value of a test answers the question: ‘How likely is it that this patient does not have the disease given that the test result is negative?’

Negative predictive value =True negatives / (True negatives + False negatives) 

**Table1:** Expression of Ki-67 & P 16 INK4a Immunostain in Cervical Biopsy According to Histologic Grading

Histologic Category	Ki-67 +ve	Ki-67 -Ve	p16 +ve	P16 -ve	Total N=110
Cervicitis (ND)	2	13	0	15	15
Metaplasia(ND)	2	8	2	8	10
CIN1	10	15	8	17	25
CIN2	11	10	11	10	21
CIN3	12	0	9	3	12
SCC	26	0	26	0	26
Adenocarcinoma	1	0	1	0	1
Total	61	49	57	53	110

ND, Nondysplasia; CIN, Cervical intraepithelial neoplasia; SCC, Squamous cell carcinoma

**Table 2:** Association Histologic Grade with of Ki-67 & P16 ^INK4a^ Singl & Dual Stain

Histology	Nondysplasia		CIN & Higher-Grade lesion		p. value
IHC marker	Positive	Negative	Positive	Negative	
Ki-67	4	21	57	28	<0.0001
P16^INK4a^	2	23	55	30	<0.0001

χ^2^-value of Ki-67 = 18.37*; χ^2^-value of P16 INK4a=24.88*; χ^2^-value of Both Ki-67 & P16 INK4a = 91.32* P value is <0.0001 [* =Significant]

**Table 3:** Diagnostic Values of Ki 67 and P16 Expression in Cervical Biopsy According Top It’s Grading

Variables	Sensitivity (%)	Specificity (%)	Positive predictive Value (%)	Negative Predictive Value (%)
Ki-67 & Routine Histology	84.00; (95%CI: 63.92-95.46)	67.06;(95% CI:56.02-76.88)	42.86; (95%CI:28.86-57.84)	93.44; (95% CI:84.07-98.18)
P16 ^INK4a^ & Routine Histology	92.00; (95%CI:73.96-99.02)	64.71 (95%CI:53.61-74.80)	43.4 (95%CI: 29.82-57.75)	96.49 (95%CI: 87.88-99.57)
Ki-67 & P16 ^INK4a^ Both stain	92.45(95%CI: 82.78-97.90)	100.00;(95%CI: 93.74-00.00)	100.00;(95%CI: 92.75-100.00)	93.44;(95%CI: 84.07-98.18)

CI, Confidence Interval

## Results

Ki-67 expression was seen in 55.5% (61/110) cases of the cervical lesion., out of which 3.6% (4/110; cervicitis -2/110 and metaplasia-2/110) cases were non-dysplasia, 51.8% (57/110) cases were dysplasia /CIN of varying grade including invasive cancer. P16 INK4a expression was noted 51.8% (57/110) (Table 1). A significant association was observed between the routine histologic grading and Ki-67 (*χ*^2^=18.37*, p<0.0001) and P16 INK4a (*χ*^2^=24.88*, p<0.0001). The combination of both immuno-histochemical stain of Ki-67 and P16^INK4a^ in cervical biopsy also showed a significant association with histologic grading of the cervical lesion. (*χ*^2^= 91.32*, p<0.0001) (Table 2) The diagnostic value of Ki-67 and P16 INK4a immuno-histochemical markers on cervical biopsy had been evaluated by calculating the Sensitivity, Specificity, Positive predictive value (PPV) and Negative Predictive Value (NPV) of the cervical lesion. The sensitivity of Ki-67 and P16 alone were 84.0% and 92.0%, respectively, and the specificity was 67.1% and 64.7% respectively. The sensitivity of the The sensitivity of both the stain together was 92.45% and the specificity was 100%. The other variables like PPV and NPV are detailed in Table 3.

## Discussion

The result of our study showed a strong association between Ki-67 and P16^INK4a^ with the grade of the cervical lesion. These markers are good indicators of high-grade CIN as reported by several authors (Halloush et al., 2008; Diouf et al., 2020; Ghosh et al., 2020). Our study also showed a significant association of Ki-67 and P16^INK4a ^in the evaluation of true precursor lesion and invasive cancer (*χ*^2^-value = 91.32* P value is <0.0001). Nam et al., (2008) found that the expression of Ki-67 (p=0.003) and P16^INK4a^ (p<0.001) were positively associated with CIN grade. Because the majority of CIN-1 lesions and a considerable number of CIN-2 lesions regress spontaneously, the ability to predict the development of CIN also is an important issue for cervical cancer prevention and treatment (Ostor,1993). The result of the present study shows a strong association of cervical neoplasia of different grades with staining pattern of P16^INK4a^ (p<0.0001) and Ki-67 (p<0.0001). These two markers are recommended as complementary tests for differentiating between dysplastic and non-dysplastic lesion by Aslani et al., (2013). The study of Yu et al., (2016) involving p16/Ki-67 co-expression showed a strong association with CIN 2 + lesion (HR-HPV persistence, especially with HPV16/18) for which p16/Ki-67 could be considered as a suitable biomarker for cervical cancer screening. Diagnosis using P16 has high specificity (94.6%), but the sensitivity is poor (85.4%). When the two were combined for diagnosis, sensitivity (94.8%) and specificity (93.2%) were both at a high level. The combined detection of Ki-67 and P16 protein has a high application prospect as an auxiliary diagnosis of SCC. (Quin Shi et al., 2019) which is similar to the present study. Ding et al., (2020) reported the importance of both P16 INK4a and Ki-67 immunostaining in the LSIL category to predict the outcome in future as their study provides new insight into identifying LSIL patients at a higher risk of malignant progression, potentially facilitating more cost-effective and efficient interventions (Ding et al., 2020). The limitation of our study is the small sample size. Due to the retrospective nature of the study, the cases could not be followed up. Another limitation is that we could not correlate the Ki-67 and P16 INK4a expression with their human papillomavirus (HPV) status. 

In summary, the expression of Ki-67 and p16^INK4a^ were seen in all the cases of cervical cancer and CIN3. The expression of both the markers was strongly associated with the degree of cervical lesions, and the sensitivity and specificity of the combination of both were satisfactory. Hence the application of Ki-67 and P16^INK4a ^ in cervical biopsy would support the histopathological features of CIN and also predict the clinical behaviour of the lesion and their possibility of progression to higher grade lesion. Their combined use may assist in the histopathologic classification of preinvasive lesions and facilitate the distinction from nondysplasia 

## Author Contribution Statement

US: Concept, laboratory testing and writing; GD: Clinical material and Data collection; BS: Statistical Analysis and language 
